# Supplementary material for: Linking mountaintop removal mining to water quality for imperiled species using satellite data
Source: PLoS One. 2021 Nov 4;16(11):e0239691. doi: 10.1371/journal.pone.0239691 (PMC8568141; doi:10.1371/journal.pone.0239691)
Supplement: Attachment — Submitted filename: Response_to_reviewers.docx [file pone.0239691.s002.docx]

**Response to reviewers**

We appreciate the time and thought put into the provided feedback by the reviewers, editors, and journal. Our point by point response to reviewer comments in the table below.

| **Reviewer** | **Comment** | **Response** |
| --- | --- | --- |
| 2 | The introduction is too long and should be shortened and made more concise | We have revisited the introduction and made revisions that have shortened the length substantially. |
| 2 | 1). Analysis of biota in contaminated and uncontaminated areas is never explicitly compared, paired tests and analysis are needed to establish a causal impact. The manuscript repeatedly states that tolerances are being exceeded, but the impacts on biota are not clearly analysed. Much of the manuscript reads as if the initial aim was to look at mining and pollution, and that biodiversity was added later to increase readership and relevance | These comments are helpful for understanding how readers may interpret the purposes and scope of this work, which we address in two parts.   First, the goals of this research are to test if there are relationships between remotely sensed data (satellite imagery) of MTR to in-stream water quality data for parameters related to wildlife health and conservation, then link any such relationships to the regulatory implications under, especially, the ESA. We believe it is unnecessary and beyond the scope of this work to have to analyze the impacts of contamination on biota for this contribution to be acceptable for publication. These results have been reported in other literature and are used by scientists and regulatory entities like those that implement the ESA. Taken to its logical end, the reviewer's comment would require that any research published not only cite other work but replicate such work before inferences can be drawn. That written, we have revisited the manuscript throughout to ensure that the scope is clear and that potentially causal relationships are conveyed appropriately.  Second, the biodiversity component of the research was first-and-foremost; two of the authors are from a wildlife-focused nonprofit, and the third from one that has a great interest in biodiversity. We have revisited the manuscript with your observation in mind and made edits to help clarify that focus. In the Introduction we make explicit our interest in assessing the potential impacts of mining on imperiled species through its effects on water quality. |
| 2 | Many more explicit details on the impacts are needed. | While it is not entirely clear which impacts the reviewer is referencing, following on our response above, we respectfully disagree that this contribution requires many more explicit details of impacts [on biodiversity] to be a contribution that advances science. As noted above, the impacts of the water quality measures we analyze to various taxa have been documented elsewhere, and we reference this literature throughout the manuscript. |
| 2 | Secondly, and following on this, better species specific analysis (i.e. by guild) are needed in relation to sensitivity, and contaminant specific analysis also needed. The main headline here is "mining contaminates rivers, this could be problematic" but explicit analysis on how species are impacted is needed. This will require extensive restructuring and further analysis to elaborate the findings of the paper. | Related to the comments above, we believe there is a misunderstanding of the goal of the present contribution. Our goal is to test the hypothesis that there is a relationship between MTR changes as detected using satellite imagery and in-stream water quality measurements in the catchments of those MTR footprints for parameters that have known effects on biodiversity, in particular ESA-listed species for which regulations have established thresholds of exposure given the best available scientific information. Our goal is not to do a meta-analysis of the effects of particular contaminants on species or the sensitivity of those species, nor to do in a single undertaking a full causal chain analysis that goes from satellite to organism response. The latter is very interesting and building on this contribution could be a subsequent step in this research program, but for our purposes, the satellite to instream measurements connection, placed in the context of ESA regulations, is sufficient. |
| 1 | 1. Introduction: In the first paragraph, the authors have reiterated about “detrimental effects of environmentally damaging activities”. I suggest the authors to provide a few examples of such damaging activities and their potential impacts to introduce the study in a more comprehensible manner. | Thank you for the suggestion; we have incorporated examples of the ways in which surface mining degrades surrounding forested ecosystems, previously in the second Introduction paragraph, into this first paragraph to provide a comprehensible example of these dynamics. |
| 1 | 2. Please rephrase the line – “Additionally, pollutants can be transferred through food webs to downstream terrestrial ecosystems (14)”. I think that the authors meant to convey that pollutants can be transferred downstream by water flow, across food webs, and into terrestrial ecosystems. | We have revised the sentence. It now reads "Additionally, pollutants can be transferred across food webs to terrestrial ecosystems." |
| 1 | 3. I suggest the authors to provide the year of enactment of the acts mentioned in the paper to provide non-native readers an idea about the timeline. | We have added the years in which the ESA and CWA were passed. |
| 1 | 4. Were any correlation tests performed to rule out correlated variables from the GLMMs? | Thank you for the question. We now describe the correlation test used to evaluate collinearity among predictor variables used in linear models at the end of the Methods, and report that these variables did not exhibit evidence of collinearity in the Results. |
| 1 | 5. The authors should provide a table of the variables used in the study. | All variables used in this study are currently listed in Tables 1 and 3. Tables 1 and 2 include all water quality measures that served as response variables in the different analyses. Table 3 provides AIC weights for each of the landscape condition variables (e.g. percent impervious, percent ag, etc.) that were used as predictor variables. |
| 1 | 6. Figure 1: Please provide a map of the USA with the highlighted study area. | We have added an inset map of the United States to this figure, which shows the larger study area. |
| PLoS | 1. Please ensure that your manuscript meets PLOS ONE's style requirements, including those for file naming. The PLOS ONE style templates can be found at https://journals.plos.org/plosone/s/file?id=wjVg/PLOSOne_formatting_sample_main_body.pdf and https://journals.plos.org/plosone/s/file?id=ba62/PLOSOne_formatting_sample_title_authors_affiliations.pdf | We have made changes throughout the manuscript to meet the requirements. |
| PLoS | 2. We noted in your submission details that a portion of your manuscript may have been presented or published elsewhere. "Yes. The mine footprint data used to estimate was previously published in Pericak A et al. (2018) Mapping the yearly extent of surface coal mining in central appalachia using landsat and Google Earth Engine. PLoS One 13(7). These data are publicly available at: https://skytruth.org/mtr-data-files/" Please clarify whether this publication was peer-reviewed and formally published. If this work was previously peer-reviewed and published, in the cover letter please provide the reason that this work does not constitute dual publication and should be included in the current manuscript. | The MTR footprint data were the focus of a paper published in PLoS One, as noted in the comment. We do not believe this could possibly be considered dual publication because the previous paper did not connect the MTR data to in-stream water quality data, which is the focus of the present contribution; we simply are using the MTR as an input dataset. We have provided an explanation to this effect in the cover letter. |
| PLoS | 3. We note that Figure 1 and Supplementary Figures F1, F2 in your submission contain map/satellite images which may be copyrighted. All PLOS content is published under the Creative Commons Attribution License (CC BY 4.0), which means that the manuscript, images, and Supporting Information files will be freely available online, and any third party is permitted to access, download, copy, distribute, and use these materials in any way, even commercially, with proper attribution. For these reasons, we cannot publish previously copyrighted maps or satellite images created using proprietary data, such as Google software (Google Maps, Street View, and Earth). For more information, see our copyright guidelines: http://journals.plos.org/plosone/s/licenses-and-copyright. We require you to either (1) present written permission from the copyright holder to publish these figures specifically under the CC BY 4.0 license, or (2) remove the figures from your submission:   3.1.    You may seek permission from the original copyright holder of Figure 1 and Supplementary Figures F1, F2 to publish the content specifically under the CC BY 4.0 license.   We require you to either (1) present written permission from the copyright holder to publish these figures specifically under the CC BY 4.0 license, or (2) remove the figures from your submission:  3.1.    You may seek permission from the original copyright holder of Figure 1 and Supplementary Figures F1, F2 to publish the content specifically under the CC BY 4.0 license.   We recommend that you contact the original copyright holder with the Content Permission Form (http://journals.plos.org/plosone/s/file?id=7c09/content-permission-form.pdf) and the following text:  “I request permission for the open-access journal PLOS ONE to publish XXX under the Creative Commons Attribution License (CCAL) CC BY 4.0 (http://creativecommons.org/licenses/by/4.0/). Please be aware that this license allows unrestricted use and distribution, even commercially, by third parties. Please reply and provide explicit written permission to publish XXX under a CC BY license and complete the attached form.”  Please upload the completed Content Permission Form or other proof of granted permissions as an "Other" file with your submission.  In the figure caption of the copyrighted figure, please include the following text: “Reprinted from [ref] under a CC BY license, with permission from [name of publisher], original copyright [original copyright year].”  3.2.    If you are unable to obtain permission from the original copyright holder to publish these figures under the CC BY 4.0 license or if the copyright holder’s requirements are incompatible with the CC BY 4.0 license, please either i) remove the figure or ii) supply a replacement figure that complies with the CC BY 4.0 license. Please check copyright information on all replacement figures and update the figure caption with source information. If applicable, please specify in the figure caption text when a figure is similar but not identical to the original image and is therefore for illustrative purposes only.  The following resources for replacing copyrighted map figures may be helpful: USGS National Map Viewer (public domain): http://viewer.nationalmap.gov/viewer/  The Gateway to Astronaut Photography of Earth (public domain): http://eol.jsc.nasa.gov/sseop/clickmap/  Maps at the CIA (public domain): https://www.cia.gov/library/publications/the-world-factbook/index.html and https://www.cia.gov/library/publications/cia-maps-publications/index.html  NASA Earth Observatory (public domain): http://earthobservatory.nasa.gov/  Landsat: http://landsat.visibleearth.nasa.gov/  USGS EROS (Earth Resources Observatory and Science (EROS) Center) (public domain): http://eros.usgs.gov/#  Natural Earth (public domain): http://www.naturalearthdata.com/ | We have replaced the basemap imagery in Figure 1 with Landsat data, which are provided publicly by the USGS and NASA. We have added language to the Figure 1 caption providing attribution to this source as recommended by NASA https://landsat.visibleearth.nasa.gov/. We do not intend to publish any supplemental figures. Those referenced, unless we are mistaken, refer to the figures contained in the study previously published in PLoS One whose output data we build upon. This document was included for review purposes to make explicit where data used in this manuscript had previously been published. We have not modified or added new legends for these figures. |
| PLoS | 4. Please include captions for your Supporting Information files at the end of your manuscript, and update any in-text citations to match accordingly. Please see our Supporting Information guidelines for more information: http://journals.plos.org/plosone/s/supporting-information. | We have updated the file name for our supplemental material, as well as all in-text references in accordance with PLoS standards. |
