# Supplementary material for: Linking mountaintop removal mining to water quality for imperiled species using satellite data
Source: PLoS One. 2021 Nov 4;16(11):e0239691. doi: 10.1371/journal.pone.0239691 (PMC8568141; doi:10.1371/journal.pone.0239691)
Supplement: Attachment — Submitted filename: Response_to_reviewers_2.docx [file pone.0239691.s003.docx]

**Editor's comments***.*

*I think this manuscript is nearly there. I do suggest that the discussion could be shortened and focussed more closely on the title. This may then deal with many of the third reviewer's comments*

We appreciate the positive feedback. In response to this and the reviewers’ suggestions we have shortened both the Introduction and Discussion. Specifically, we narrow and limit the scope of our discussion of conservations laws and regulations, except to make direct connections to the ways in which the results of this analysis directly inform their implementation.

**Reviewer #1:**

*I have checked this paper earlier. The paper is now ready for acceptance. I have no further comment.*

We appreciate the repeated review and help in shaping this manuscript for publication, and are happy to hear that we were able to address the previous suggestions.

**Reviewer #3:**

*The water quality data show that chronic and acute thresholds for aquatic life were exceeded thousands of times between 1985 and 2015 in streams that are important to the survival and recovery of species on the Endangered Species List. {which parameters? Name these parameters]*

As suggested, we have included the names of each of the parameters for which chronic and acute aquatic life thresholds were exceeded: Aluminum; Arsenic; Cadmium; Conductivity; Copper; Lead; Manganese; Mercury; pH; Selenium; and Zinc

*Linear mixed models showed that levels of conductivity, manganese, sulfate, sulfur, total dissolved solids, total suspended solids, and zinc increased as the proportion of the area draining into a monitoring station that was mined increased. [mention the increase as %]*

We have included the range of beta coefficients for these analyses in this sentence, presenting the average increase in each measure for a one percent increase in mined area. The sentence now reads:

“Linear mixed models showed that levels of conductivity, manganese, sulfate, sulfur, total dissolved solids, total suspended solids, and zinc increased by 6.73E+01 to 3.30E+06 for one percent increase in the mined proportion of the area draining into a monitoring station.”

*Introduction*:
*The release of alkaline mine drainage from weathering of rock and site waste at mine sites elevates conductivity and concentrations of metallic ions that negatively impact aquatic biota [12], leading to decreases in aquatic biodiversity [10,13]*

As far as we can tell, this sentence is identical to what is currently in the manuscript. If the reviewer is suggesting any changes it is unclear what they are.

*Introduction part is very lengthy, it can be reduced substantially (at least 30-40%)*

We have condensed the two Introduction paragraphs describing the legal and regulatory context in this work into a single paragraph. This aligns with a shortening of the Discussion aimed at focusing the text more closely on the results of this study. Additionally, we have made reductions in the length of the Introduction by eliminating and shortening sentences throughout.

*Methods: section needs through rewriting and Cleary mention commensurate with objectives of study.*

We have edited several sentences that were flagged as confusing or lacking clarity below, as well as re-organized the order in which the Table 1 is presented relative to text describing its contents.

*This portion is not clear. Be specific, for examples, under “Water Quality data” -- .*

We are not entirely clear as to what this comment pertains, but have re-read the Methods section for clarity, rearranging sections to improve the logical presentation of the data collection and analyses.

*Additionally, we collected flow rate and temperature data. # where it has been used? And how it has been used?*

We initially collected these variables for potential use in linear mixed models. We have amended this sentence acknowledging that these variables were not used. Additionally, in the following section we omit describing the conversion of temperature to degrees Celsius.

*Table 1. Chronic and acute toxicity thresholds were exceeded many times in waterways important to listed aquatic species [NOT CLEAR?], For examples, Arsenic 39 (chronic) 39 (acute), what are these values? Is that concentration?? Or what? Not Clearly explained in the text.*

These values are counts representing the number of times a recorded water quality measure exceeded standard thresholds for aquatic life, and thus are unitless. This description is provided in the Table footer:

“Table shows the number of times that any recorded value (e.g. ‘Dissolved’, ‘Total’, etc.) of each water quality measure exceeded standard thresholds for aquatic life, and the number of different monitoring stations at which these events occurred. These data only consider measures taken from 1,887 monitoring stations whose drainage basin contained a stream that was designated as important to species survival and recovery.

^a^No chronic or acute thresholds provided for measure”

Thresholds are not species specific, and we describe their origin in the Methods section as follows:

“To identify acute and chronic toxicity thresholds for different water quality measures, we used the state water quality standards for aquatic life administered by Virginia under the Clean Water Act. These standards are approved by the Environmental Protection Agency and are used to determine waterway impairment requiring mitigation. The water quality standards thus represent an agreed upon set of thresholds necessary to maintain suitability of waterways for aquatic species. The Virginia standards were identical to those from Kentucky, Tennessee and West Virginia.”

We have added a citation for the Vriginia Clean Water Act standards. We have moved the location of Table 1 to follow this description so that the context of the data presented in the table is more clear.

*# Water quality data were provided in different units, and we standardized all measures of concentration to ug/L, temperature to degrees Celsius, conductivity to µS /cm, and turbidity to NTU.
\## wright inside the table for better clarity.*

We have added units to Table 2, which presents regression coefficients estimating the scale of increase or decrease in each water quality measure. As noted above, Table 1 reports the frequency with which thresholds for aquatic life established for each measure were exceeded. Thus, we omit units here.

*For Zinc 451(ug/L) for Acute and 451 (ug/L) for chronic. PLEASE CHECK THE DATA from the sources, where it has been collected? How concentration of Zn (451 ug/L) will be same for chronic and acute toxicity??*

Assuming these comments refer to Table 1, the reviewer has misunderstood the data being presented in the table, which consists of counts and not concentrations. We have moved the location of the table to the end of the Methods section, to provide more complete context for these data.

RESULTS
*From m 0 to 343 km (x = 77.7 km, σ 2 = 4071 km), check data*

We have double checked our data and confirm this statement is correct. We have modified how we present the results, as 0 km may cause confusion. We now state that:

“Distances between stations and mines were exponentially distributed, ranging from < 1 to 343 km (x = 77.7 km, σ^2^ = 4,071 km).”

*# The number of observations at each site ranged from 1 to 275. What is the meaning?*

We have added language clarifying that an observation refers to an occasion on which water quality data was recorded at a site. The sentence now reads:

‘The number of observations (i.e., occasions on which water quality data was recorded) at each site ranged from 1 to 275.’

*Table 2: Total dissolved solids Total
Total dissolved solids Dissolved* (* implies dissolved?)
Total suspended solids Total
Check the solids nomenclature,*

These are the measures provided by the national water quality data portal, as recorded by the U.S. Geological Survey, National Park Service, and other state and federal agencies.

*Conclusions*
*The conclusions must be based on the study undertaken. Entire conclusions discussed impacts of mining on imperiled species through degradation of water quality. It that is so, then in results section provide some tables which are now added as supplementary materials.*

We have reduced the length of the Discussion by truncating sections less directly related to the study at hand. Specifically we shorten our discussion of SMCRA and the regulatory obligations of the Fish and Wildlife Service under section 7 of the Endangered Species Act.

We mirror these changes in the Conclusion, although retain a focus on placing the findings of this study in a broader conservation and regulatory context. We have edited language to connect the outcomes of this study more clearly to these broader conclusions.

Overall comments:
*There has been nobility in this approach, and could be useful, but methods & results are not clear. It is more of secondary data used in a statistical tools. In such cases, a flow chart should be provided for meaning full interpretation of existing data.*

Previous reviewers and editors have not had challenges understanding the study design, use or analysis of data. In response to this comment, we have invited a colleague unfamiliar with the project to evaluate the manuscript for clarity and interpretability. They did not identify any major barriers to understanding the methodology and analytical approach, beyond clarifying some of the points regarding units and measures raised above. At this point, we are not inclined to provide a flow chart as an additional figure, and believe the changes made to the Methods and Results are sufficient to provide a clear interpretation of how the data were used and analyzed.

*I have a strong reservation for recommending this paper for publication, however, this article may be again rewrite and resubmit.*

We appreciate the feedback provided and believe we have been able to address all current and previously raised concerns.
